# Adolescent motherhood in Mozambique. Consequences for pregnant women and newborns

**DOI:** 10.1371/journal.pone.0233985

**Published:** 2020-06-03

**Authors:** Nieves Jaén-Sánchez, Gloria González-Azpeitia, Pedro Saavedra-Santana, Esther Saavedra-Sanjuán, Aniceto-Alberto Manguiza, Nicholas Manwere, Cristina Carranza-Rodriguez, José Luis Pérez-Arellano, Lluis Serra-Majem

**Affiliations:** 1 Infectious Diseases and Tropical Medicine Division, Complejo Hospitalario Univer-sitario Insular Materno Infantil, Las Palmas de Gran Canaria, Spain; 2 University of Las Palmas de Gran Canaria, Las Palmas de Gran Canaria, Spain; 3 Pediatric Division, Complejo Hospitalario Universitario Insular Materno Infantil, Las Palmas de Gran Canaria, Spain; 4 Department of Clinical Sciences (iUIBS), Research Institute of Biomedical and Health Sciences, University of Las Palmas de Gran Canaria, Las Palmas de Gran Canaria, Spain; 5 Department of Mathematics, University of Las Palmas de Gran Canaria, Las Palmas de Gran Canaria, Spain; 6 University of Zambeze, Beira, Mozambique; 7 Ciber OBN (CB06/03), Instituto Carlos III, Spanish Government, Madrid, Spain; University of Oxford, UNITED KINGDOM

## Abstract

**Introduction:**

In sub-Saharan Mozambique, high adolescent fertility rates are a significant public health problem. Understanding the consequences of teenage pregnancies facilitates effective strategies for improving the quality of care of both mother and the newborn.

**Aims:**

To identify the factors associated with adolescent motherhood in Tete (Mozambique).

**Methods:**

This was a cross-sectional study including 821 pregnant women (255 teenagers) admitted to the general maternity ward of the Provincial Hospital between March and October 2016. The survey included clinical data of the mother and newborn.

**Results:**

The overall prevalence of adolescent deliveries was 31.8% (95% CI 27.9% - 34.2%). Multivariate analysis showed that independent factors associated with teenage motherhood were: number of pregnancies (OR 0.066; 95% CI 0.040–0.110), pregnancy follow-up (OR 0.29; CI 0.173–0.488) and previous abortions (OR 4.419; 95% CI 1.931–10.112). When the age of the mother was analysed as a continuous variable, positively associated factors were body mass index, arterial hypertension, HIV infection, previous abortions, pregnancy follow-up, and the weight of the newborn. Negatively associated factors were episiotomy and respiratory distress in the newborn.

**Conclusion:**

Teenage motherhood is a serious public health problem in Mozambique. Intensive sexual and reproductive health planning for adolescents is needed.

## Background

The adolescent population includes individuals in the 10–19 years group [1], representing those in the transitional phase between childhood and adulthood. This life period is marked by intense physical, psychological, emotional, and economic changes [1, 2]. In many cultures, this developmental phase does not exist or is relatively brief, particularly when controlled by initiation rites [3].

Approximately 16 million girls aged 15–19 years and 2.5 million girls under 16 years of age give birth every year in developing regions [4]. The maternal mortality rate among girls aged 15–19 years in low- and middle-income countries (LMICs) in the African region is very high (36 per 100,000 population), followed by 9, 7, and 3 deaths per 100,000 in the LMICs of the Eastern Mediterranean, South-East Asia and the Americas region, respectively [1].

Interventions to reduce the prevalence of adolescent pregnancies in developing countries is therefore a public health imperative, aimed at achieving sustainable development goals by 2030 [5].

In Mozambique, 38% of adolescent girls have given birth to a live child. This is the highest adolescent fertility rate in the countries of the Southern African Development Community, with a rising trend between 1997 and 2015 [6]. Furthermore, Mozambique is one of six countries in the world where at least one in ten girls (14%) has had a child before the age of 15, and 57% before age 18 [6, 7]. Mozambique has the 10th highest rate of child marriage in the world, measured as the proportion of women aged 20–24 who married in childhood (under 18 years old). Rates of child marriage are much higher than the averages in Eastern and Southern African sub-regions and are exceeded only by another Southern African country, Malawi [8]. According to data from the Demographic and Health Survey (DHS), in 2011 48% of women aged 20–24 were married before the age of 18, and 14% even before the age of 15 [9].

Both mother and child are at increased risk of adverse outcomes in adolescent pregnancies [10, 11]. There is also a high probability that the young mother will interrupt her education or drop out of school altogether, increasing the risk of lower socio-economic status, poverty, and even death from pregnancy-related complications [12].

In many African countries, it is common for people to be born, grow up, and die without being officially registered. In Africa, only Mauritius and the Seychelles have complete registrations of births, deaths, and causes of death [13] (the data from the most recent survey published in Mozambique indicates that childbirth increased from 48% to 55% between 2011 and 2015) [6].

Reliable statistics on maternal and perinatal morbidity and mortality are scarce in low- and middle-income countries, particularly in rural areas like Mozambique [14]. This information is crucial for developing effective national and global health policies concerning maternal and child health.

## Data and methods

### Study setting and participants

**Fig 1:**
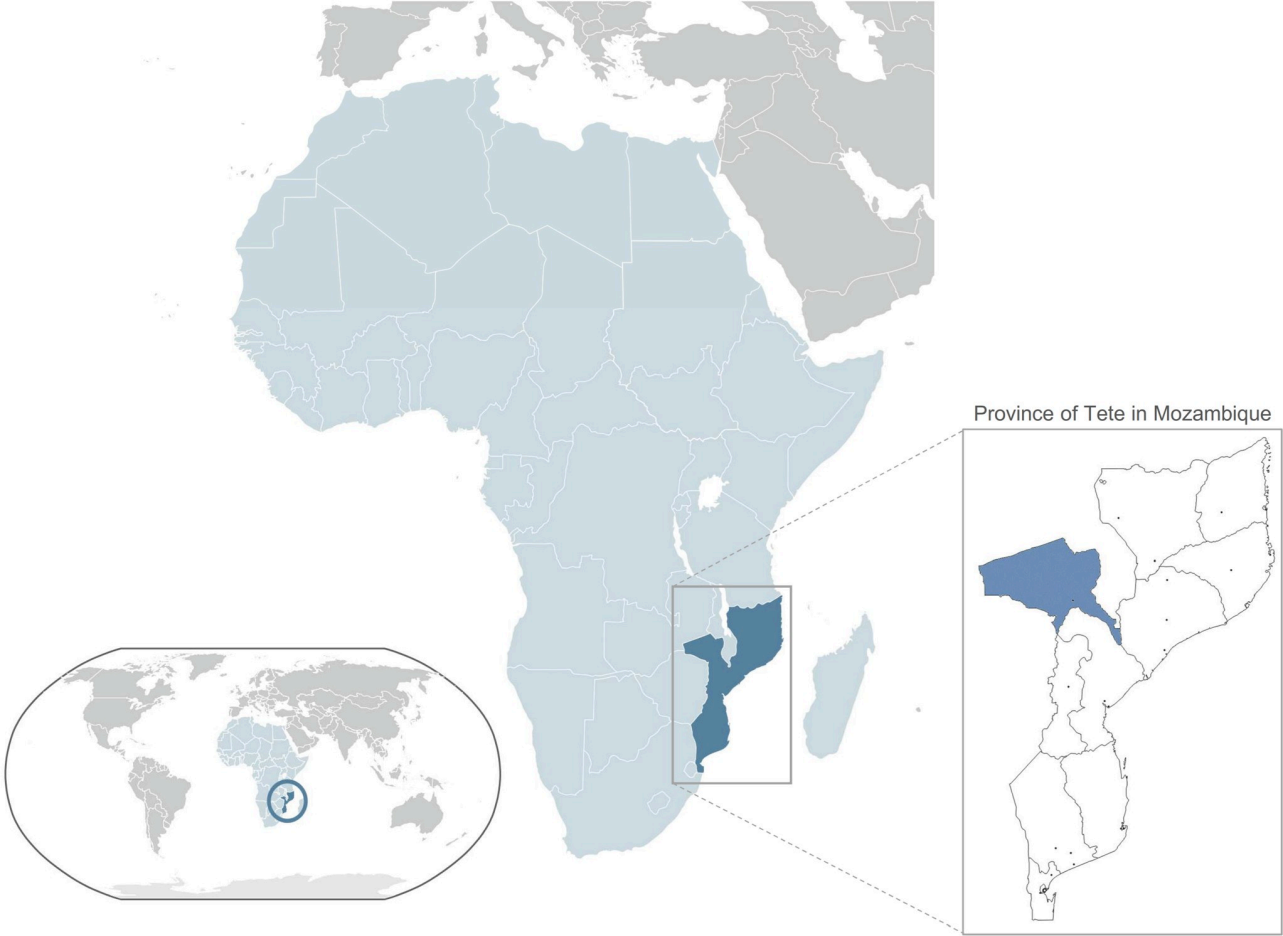
Geographical situation of the study area.

**Fig 2:**
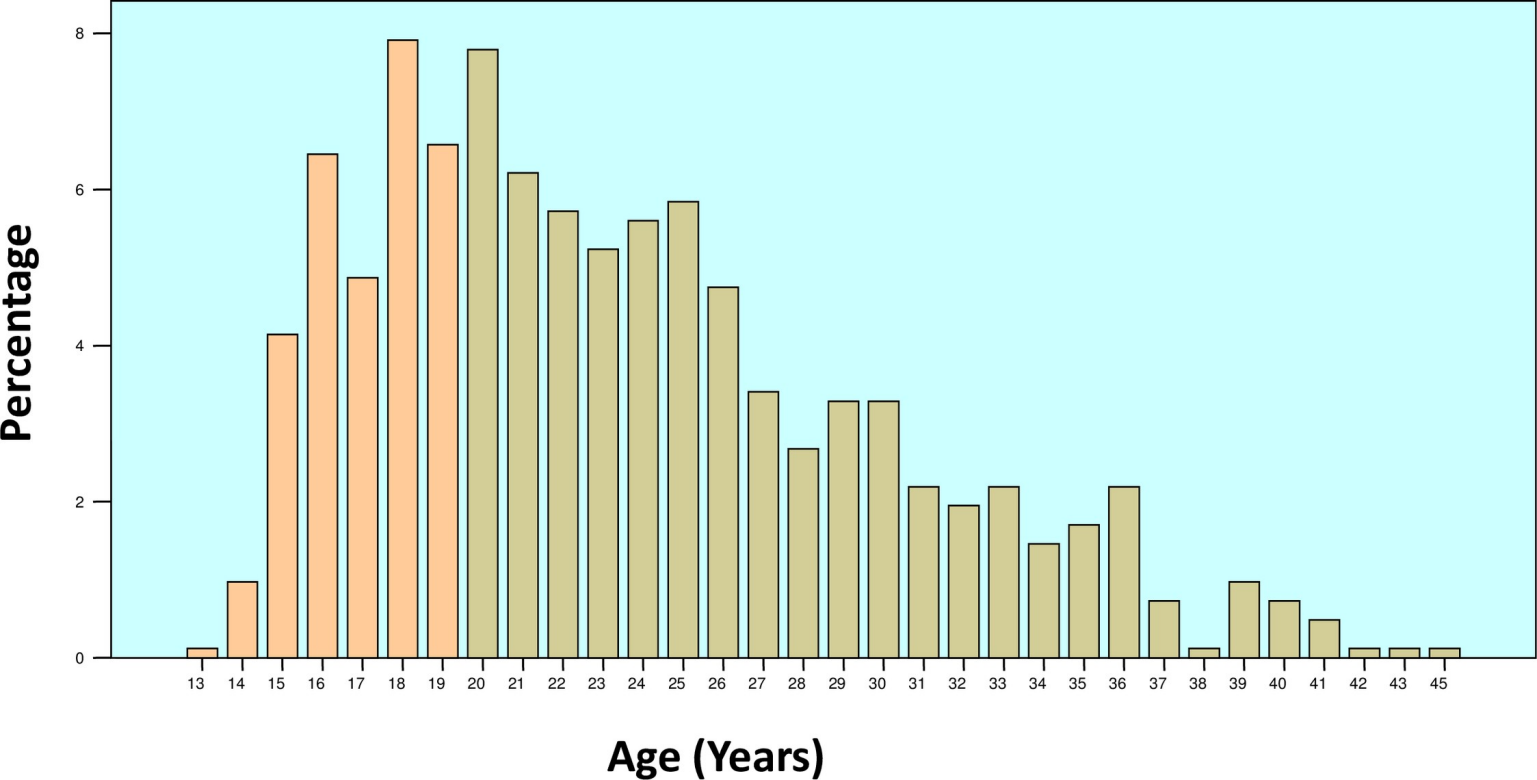
Distribution of study subjects according to their age.

The data was collected from the maternity department of the Provincial Hospital of Tete (PHT), between March and October 2016. The province of Tete is located in the central region of Mozambique sharing a border with Zambia to the northwest and Zimbabwe to the southwest (Fig 1). According to the 2017 National Institute of Statistics Annual Report for 2017, the population of the province of Tete was 2,764,169 and 13.6% lived in urban areas [15]. The number of births during this period was 2,906. Eight hundred and twenty-one women aged between 13 and 45 years from different districts in Tete province were randomly selected (Fig 2).

### Study procedures

To minimize errors, the person responsible for collecting the data and a supervisor took part in a two-day training session focused on aspects associated with the ques-tionnaire, physical examination, and anthropometric measurements. The data was collected by completing a survey based on the pregnancy chart, which included personal details, as well as the obstetric history of the women included in the study, with information on the current gestation, events associated with the delivery, and data about the newborn.

### Study design

This was a cross-sectional study that included 821 pregnant women living in Tete (Mozambique), 255 of whom were teenagers. The subject was considered a teenager if she was under 20 years of age.

### Variables

The following variables were included in this study: age, prenatal follow-up, anthropometric measurements, such as body mass index, arterial hypertension, HIV or syphilis infection, and pregnancy and intrapartum variables. Data about the newborn was also collected, including weight, Apgar score, neonatal respiratory distress (NRD), and mortality.

### Statistical analysis

Categorical variables were expressed as frequencies and percentages, and continuous variables as means and standard deviation (SD) when the data was normally distributed, and as median and interquartile range (IQR = 25th– 75th percentile) when the data was not normally distributed. Percentages were compared using the chi-square (χ2) test or the Fisher’s exact test as appropriate; the t-test was used to compare means, and the Mann-Whitney test was used for independent data.

Multivariate binomial regression analysis was performed to identify factors independently associated with teenage motherhood. Variables significantly associated with the outcome (teenage motherhood) in univariate analysis were entered into multivariate logistic analysis. Variable selection was based on the best subset regresson and Akaike Information Criterion (AIC) was then performed. The model was summarized as coefficients (SE), p-values and odds ratios, estimated using a 95% confidence interval.

Additive models. We performed a multivariate analysis for the age of the pregnant. We carried out a selection of variables The modes were summarized as coefficients (SE). Data were analysed using the R package, version 3.3.1 (R Development Core Team, 2019) [16].

### Ethics

The study was conducted according to the criteria established by the Declaration of Helsinki [17], and approved by the National Committee for Bioethics in Health of Mozambique´s Ministry of Health. An interview was conducted only if the respondent provided their verbal consent in response to being read out an informed consent statement by the interviewer. Eligible participants provided written informed consent, which was documented in writing or with a fingerprint and witness signature prior to beginning the survey. The consent was signed by parent/guardian if the participant is under 18 years of age.

The authors declare that there are no conflicts of interest with respect to this study.

## Results

The percentage of adolescents giving birth was 31.8% (95% CI 27.9% - 34.2%). Body mass index (Table 1), was significantly lower in adolescent mothers (*p* = 0.001).

**Table 1:** Characteristics of the mothers.

		*Teenage motherhood*		
	*Overall N = 821*	*No N = 566*	*Yes N = 255*	*p*
*Age*, years	23.5 ± 6.3	26.5 ± 5.4	17.1 ± 1.5	< .001
*Body mass index*, kg/m^2^	24.4 ± 4.2	25.0 ± 4.4	23.2 ± 3.7	0.001
*Living area*				0.280
Urban	669 (89.0)	466 (90.0)	203 (86.8)	
Peri-urban	35 (4.7)	20 (3.9)	15 (6.4)	
Rural	48 (6.4)	32 (6.2)	16 (6.8)	
*Number of pregnancies*	2 (1;3)	3 (2;4)	1 (1;1)	< .001
*Previous abortion*	134 (16.3)	117 (20.7)	17 (6.7)	< .001

Data are presented as means ± SD and frequencies (%)

Teenage mothers had fewer previous pregnancies and abortions (*p* < 0.001 in both cases) (Table 1). However, these data were reversed when women were pregnant with their second child, pregnant teenage had suffered more abortions (39.5%) than adult women (13.6%, p < 0.001). Follow-up of pregnancy (Table 2) was significantly lower among adolescents (42.6% vs 63.0%; p < 0.001) and HIV infection (p < 0.001) (Table 2).

**Table 2:** Characteristics of the pregnancies and deliveries.

		*Teenage motherhood*		
	Overall = 821	No N = 566	Yes N = 255	*p*
*Pregnancy follow-up*	348 (56.9)	271 (63.0)	78 (42.6)	< .001
*Malaria during pregnancy*	92 (11.2)	64 (11.3)	28 (11.0)	0.899
*Syphilis*	9 (1.1)	6 (1.1)	3 (1.2)	1
*HIV infection*	101 (12.5)	89 (16.0)	12 (4.8)	< .001
*Systolic blood pressure*, mmHg, mmHg	125 (117;139)	124 (118;138)	128 (117;140)	0.236
*Diastolic blood pressure*, mmHg, mmHg	80 (70;90)	80 (70;90)	80 (70;90)	0.697
*Malaria at delivery*	15 (1.8)	9 (1.6)	6 (2.4)	0.574
*Placental abruption*	32 (3.9)	23 (4.1)	9 (3.6)	0.731
*Peripartum Urinary Tract Infection*	48 (5.9)	36 (6.4)	12 (4.7)	0.351
*Maternal fever at delivery*	33 (4.1)	23 (4.1)	10 (4.0)	0.930
*Intra-partum hemorrhage*	101 (13.2)	76 (14.5)	25 (10.3)	0.108
*Episiotomy*	32 (6.9)	11 (3.4)	21 (14.7)	< .001
*Hypertensive disorders of pregnancy*				0.255
Normal	697 (84.9)	483 (85.3)	214 (83.9)	
Preeclampsia	84 (10.2)	60 (10.6)	24 (9.4)	
Eclampsia	40 (4.9)	23 (4.1)	17 (6.7)	
*Gestation*				0.588
Pre-term	107 (13.2)	72 (12.9)	35 (13.9)	
Post-term	9 (1.1)	5 (0.9)	4 (1.6)	
At term	694 (85.7)	481 (86.2)	213 (84.5)	
*Gestation type*				0.080
Single	793 (96.8)	543 (96.1)	250 (98.4)	
Multiple	26 (3.2)	22 (3.9)	4 (1.6)	
*Type of delivery*				0.349
Eutocic	555 (68.0)	374 (66.5)	181 (71.3)	
Caesarean section	235 (28.8)	168 (29.9)	67 (26.4)	
Vacuum	26 (3.2)	20 (3.6)	6 (2.4)	

Data are presented as means ± SD and frequencies (%)

With respect to the newborns, the weights and first-minute Apgar score of those born to teenage mothers were significantly lower (p<0.001, p = 0.047 respectively) than those born to older mothers. Neonatal respiratory distress was more frequently detected in the babies of adolescent mothers (*p* = 0.007), regardless of gestational age (Table 3).

**Table 3:** Characteristics of the newborns.

		*Teenage motherhood*		
	Overall N = 821	No N = 566	Yes N = 255	*p*
*Sex male*	252 (44.1)	185 (46.7)	67 (38.1)	0.054
*Weight at birth*, kg	3.0 (2.6; 3.3)	3.0 (2.7; 3.3)	2.9 (2.6; 3.1)	< .001
*Height at birth*, cm	47 (43; 49)	47 (43; 49)	47 (43; 49)	0.839
*Cephalic perimeter at birth*, cm	33 (32; 35)	33 (32; 35)	33 (32; 34)	0.994
*One-minute Apgar score*	9 (8; 9)	9 (8; 9)	8 (7; 9)	0.047
*Respiratory distress*	48 (6.2)	25 (4.6)	23 (9.7)	0.007
*Death*	60 (7.3)	39 (6.9)	21 (8.2)	0.493

Data are presented as medians (IQR) and frequencies (%)

Multivariate analysis found that being a teenage mother was statistically significantly associated with having had fewer pregnancies (OR per unit = 0.057; 95% CI = 0.033–0.098), receiving less prenatal follow-up (OR = 0.290; 95% CI = 0.173–0.488) and being at greater risk of miscarriage (OR = 5.889; 95%CI = 2.365–14.661) (Table 4).

**Table 4:** Multivariate binomial regression for the teenage motherhood.

	Coefficient (SE)	*p*	OR (95% CI)
(Intercept)	1.638(0.211)	< .001	
*Number of pregnancies*, per subject	-1.805 (0.204)	< .001	0.164 (0.110; 0.245)
*Previous abortion*	1.286 (0.312)	< .001	3.617 (1.962; 6.667)
*Pregnancy follow-up*	-0.354 (0.088)	< .001	0.702 (0.591; 0.833)

**Fig 3:**
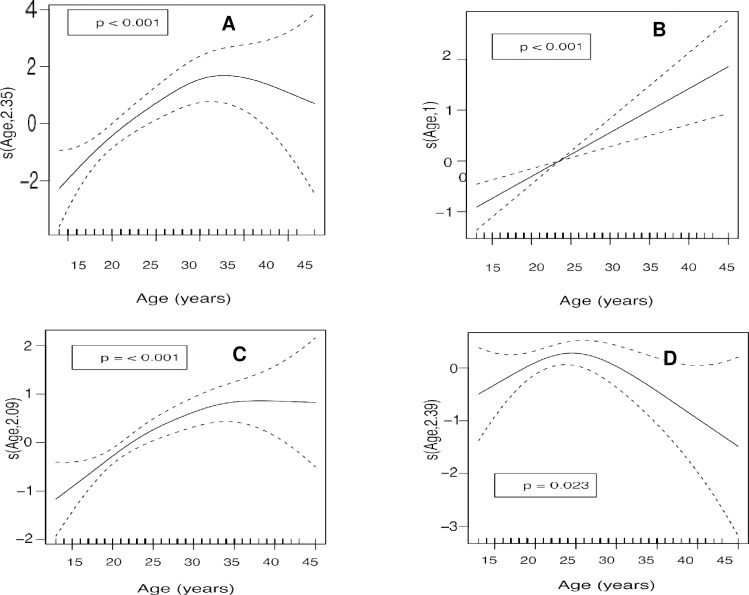
Analysis of the age of the mother versus A) Body mass index, B) Arterial hypertension, C) HIV infection, D) Previous abortions. Adjusted by the number of gestation.

**Fig 4:**
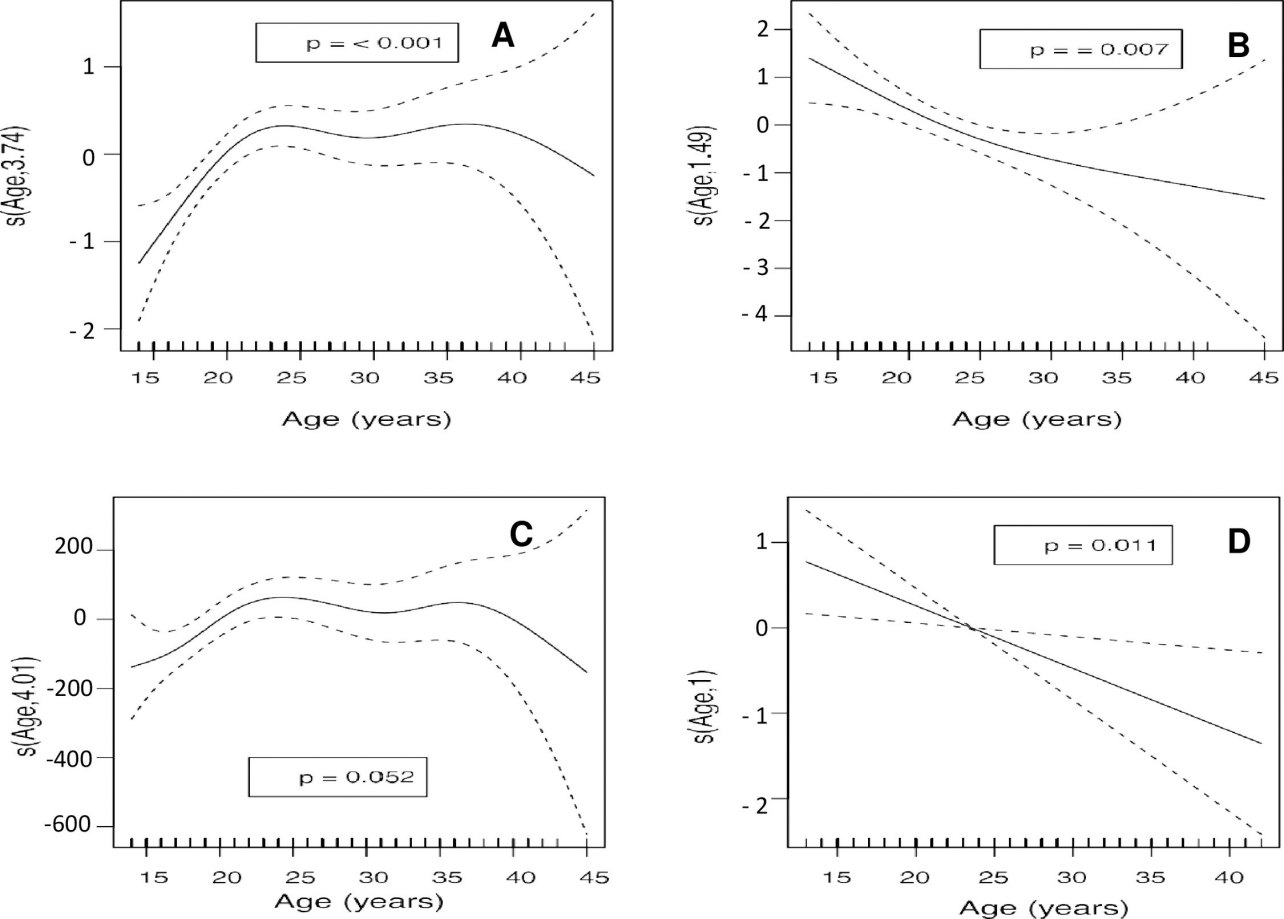
Analysis of the age of the mother versus A) follow-up of the pregnancy, B) episiotomy, C) weight of the newborn. Adjusted to the gestation week, D) neonatal respiratory distress.

An analysis of the age of the mother as a continuous variable (Table 5) revealed that BMI (Fig 3A) was low in the early years of adolescence and then increased until the mother was around 32 years of age (*p* < 0.001). Arterial hypertension (Fig 3B) was observed to increase with the age of the mother (*p* < 0.001). Prevalence of HIV infection (Fig 3C) was low at younger ages, increased until age 35, and then stabilized (*p* < 0.001). The abortion rate (Fig 3D) rose from the early years of adolescence until age 25, when it declined sharply (*p* = 0.023). Follow-up during pregnancy (Fig 4A) was less common among adolescents but increased significantly with age until around 23 years, when it stabilized (*p* < 0.001). A quasi-linear progression was observed between episiotomy rate and age (Fig 4B), being very frequent among younger mothers, then decreasing steadily with age (*p* = 0.007). The mean weight of the newborn (Fig 4C) was very low in the younger mothers and increased until age 23, at which point a decrease in mean weight was observed until the age of 30, when another increase was observed until the mother reached 35 (*p* = 0.052). Neonatal respiratory distress showed a linear decrease (Fig 4D) and was most frequently observed in younger mothers, decreasing with age (*p* = 0.011).

**Table 5:** Additive models for the effects of the age on several factors.

Dependent variable	*p-*value	Co-variable
*Body mass index*^a^, Kg/m^2^	< 0.001	None
*Arterial hypertension*^b^	< 0.001	None
*HIV infection*^b^	< 0.001	None
*Previous abortions*^b^	0.023	*Number of pregnancies*
*Control of pregnancy*^b^	< 0.001	None
*Episiotomy*^b^	0.007	None
*Weight of newborn*^a^, Kg	0.052	*Gestation week*
*Respiratory distress*^b^	0.011	None

^a^For continuous dependent variables, data were fitted by an ordinary additive model.

^b^For binary dependent variables, data were fitted by a logistic additive model.

## Discussion

In Mozambique, adolescents are the fastest growing segment of the population [18]. The fertility rate in 2015 was 5.3 children per woman and only 27% of these used some type of family planning method. Compared with currently married women, almost twice the percentage of those who were unmarried and sexually active used some family planning method [6]. Twenty-five percent of married women used modern methods and two percent traditional methods. The most commonly method used by unmarried, sexually-active women was male condoms, followed by contraceptive injection and oral [6].

Mozambique has the highest adolescent fertility rate of all the countries in the Southern African Development Community [3, 6, 7]. The 2015 IMASIDA survey indica-ted that 38% of adolescents had a child and, in the province of Tete in particular, the incidence was 46% [6].

There is a significant association between adolescent pregnancy and lower BMI and follow-up during pregnancy, similar to that reported in other publications with sub-Saharan African populations [19, 20]. Although the median weight of the newborns was significantly lower, there were more episiotomies and lower first-minute Apgar scores.

The percentage of previous abortions among adolescents in the Provincial Hospital of Tete was slightly higher than that reported in the Demographic and Health Survey (DHS) 2011 [9]. In this sample, although previous abortions among adolescents were significantly lower, after adjusting for number of gestations, the rate of previous abortions in second pregnant women was significatively higher compared with adult women.

According to the data from the last national survey carried out in Mozambique, 93% of pregnant women had one pre-natal visit and 55% had four or more [6]. However, in our study, 63% of adult women received follow-up during pregnancy (minimum four visits per pregnancy) and only 42.6% of adolescents [21, 22]. This rate of follow-up during pregnancy in Mozambique correlates with increased maternal mortality as cause of death; 24% of adolescent deaths are associated with maternity, declining to 16% in women aged 25–29, and 8% in women aged 45–49 [3, 23].

Sub-Saharan Africa remains the region with the highest percentage of pregnant women living with HIV, accounting for nearly 85% of the global burden of HIV [24]. According to UNAIDS 2017, the estimated prevalence of HIV among people aged 15–49 is 12.5% [25]. In this study, 12.5% of women sampled were HIV-positive, which is twice the 2015 number reported by IMASIDA (5.2%) for the province of Tete [6]. As in other areas of the country, the prevalence of HIV is significantly correlated with the age of the mother; the prevalence in adolescence is significantly lower, increases until age 35 and then stabilizes although in other areas of the country it decreases [26].

Caesarean section rates are used as indicators of the availability and use of life-saving obstetric services in developing countries. In the PHT, the percentage of caesarean sections was 28.8% with no significant differences in adolescents. The proportion of caesarean sections is higher than that published for public hospitals in the southern area of Mozambique between 2009 and 2011, when the highest percentage was 20.6% [27].

The incidence of preterm births in this sample was slightly lower than the figure reported in another publication on Mozambique, which pointed out that it was in the top 10 of countries in the world with the highest rates of preterm births [28]. Unlike other publications, no significant increases were observed in pre-term births or neonatal mortality in pregnant adolescents [21, 22].

The main limitations of this study are the underestimation of the prevalence of adolescent pregnancies in the central region of Mozambique and that little attention has been paid to sociodemographic factors in adolescent pregnancies [29, 30].

As an example of the reflection of this data The Mozambican Association for Family Development (AMODEFA) has a clinic that offers sexual and reproductive health services, including safe abortions. Unpublished AMODEFA data indicate that 70,895 women underwent induced abortions in their clinic between 2010 and 2016, and that 43% of these women were between 15 and 24 years old. Of the 1,500 women who had induced abortions in the AMODEFA clinic in the first three months of 2017, 27.9% were also in this age group [29, 30]. These data are indicative of the high demand for (safe) abortions among young women in the country.

In summary, this analysis highlights some of the immediate challenges facing Mozambique, trade-and emphasizes the need to improve more sexual and reproductive health services for adolescents. [31].
